# Supplementary material for: Cathodoluminescent and Characteristic X‐Ray‐Emissive Rare‐Earth‐Doped Core/Shell Protein Labels for Spectromicroscopic Analysis of Cell Surface Receptors
Source: Small. 2024 Sep 9;20(48):2404309. doi: 10.1002/smll.202404309 (PMC11600707; doi:10.1002/smll.202404309)
Supplement: Supplementary file 1 — Supporting Information [file SMLL-20-2404309-s001.pdf]

NANO · MICRO
small

Supporting Information

for *Small*, DOI 10.1002/smll.202404309

Cathodoluminescent and Characteristic X-Ray-Emissive Rare-Earth-Doped Core/Shell Protein Labels for Spectromicroscopic Analysis of Cell Surface Receptors

*Sebastian Habermann, Lukas R. H. Gerken, Mathieu Kociak, Christian Monachon, Vera M. Kissling, Alexander Gogos and Inge K. Herrmann**

Supplementary Information: Cathodoluminescent and Characteristic X-ray-emissive Rare-Earth-doped Core/Shell Immunolabels for Spectromicroscopic Analysis of Cell Surface Receptors

Sebastian Habermann, Lukas R. H. Gerken, Mathieu Kociak, Christian Monachon, Vera M. Kissling, Alexander Gogos and Inge K. Herrmann*

S. Habermann, Dr. L.R.H. Gerken, Dr. A. Gogos, Prof. Dr. I.K. Herrmann
Nanoparticle Systems Engineering Laboratory, Institute of Energy and Process Engineering, Department of Mechanical and Process Engineering, ETH Zurich, Sonneggstrasse 3, 8092 Zurich, Switzerland.
Email Address: inge.herrmann@empa.ch; ingeh@ethz.ch; inge.Herrmann@balgrist.ch

S. Habermann, Dr. L. R. H. Gerken, Dr. A. Gogos, V. M. Kissling, Prof. Dr. I. K. Herrmann
Laboratory for Particles Biology Interactions, Department Materials Meet Life, Swiss Federal Laboratories for Materials Science and Technology (Empa), Lerchenfeldstrasse 5, 9014 St. Gallen, Switzerland.

Dr. M. Kociak
Université Paris-Saclay, CNRS, Laboratoire de Physique des Solides, Orsay 91405, France.

Dr. C. Monachon
Attolight AG, 1015 Lausanne, Switzerland.

Prof. Dr. I. K. Herrmann*
The Ingenuity Lab, University Hospital Balgrist, Balgrist Campus, Forchstrasse 340, 8008 Zurich, Switzerland.

Prof. Dr. I. K. Herrmann*
Faculty of Medicine, University of Zurich, Rämistrasse 74, 8006 Zurich, Switzerland.

Keywords: Nanoparticle, EDX, Ultrastructure, Immunotargeting, Multi-Color

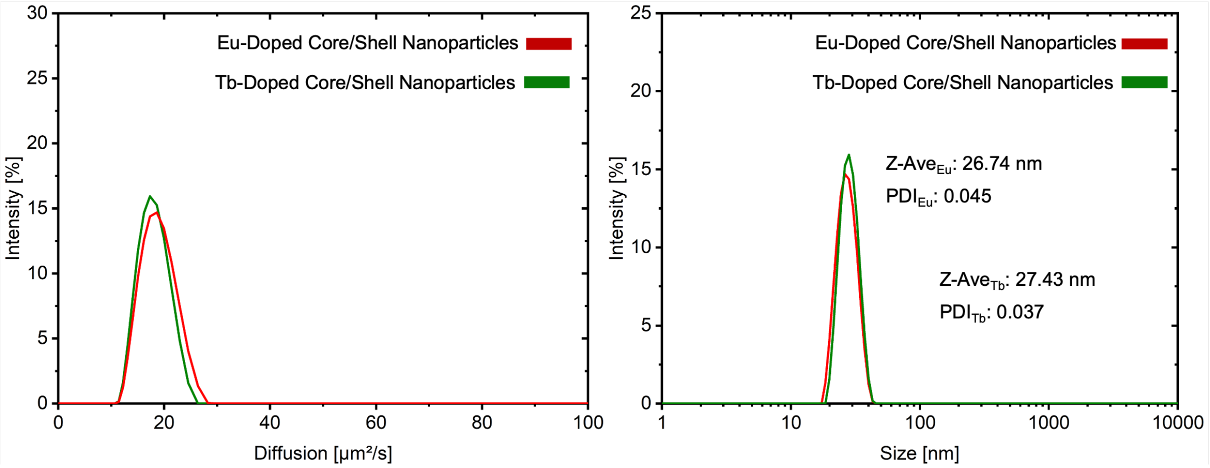

Suppl. Figure S1: Diffusional analysis of as-prepared core/shell nanoparticles, revealing an exceptionally narrow size distribution, which foreshadowed nearly identical diffusion properties.

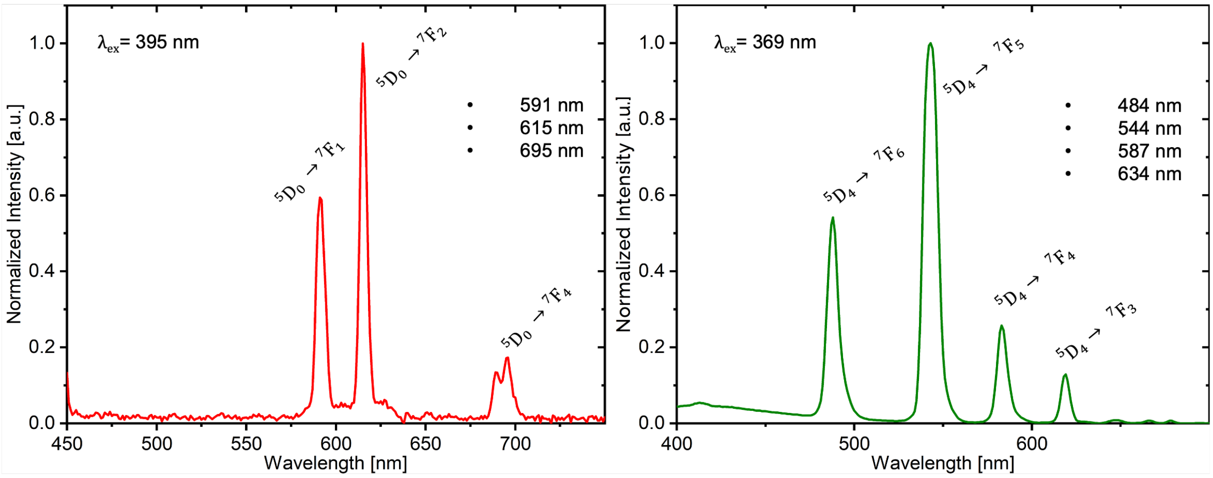

Suppl. Figure S2: Photoluminescence spectra of Eu^{3+}/Tb^{3+}-doped core/shell $NaGdF_4$ nanoparticles. a.u.: arbitrary units.

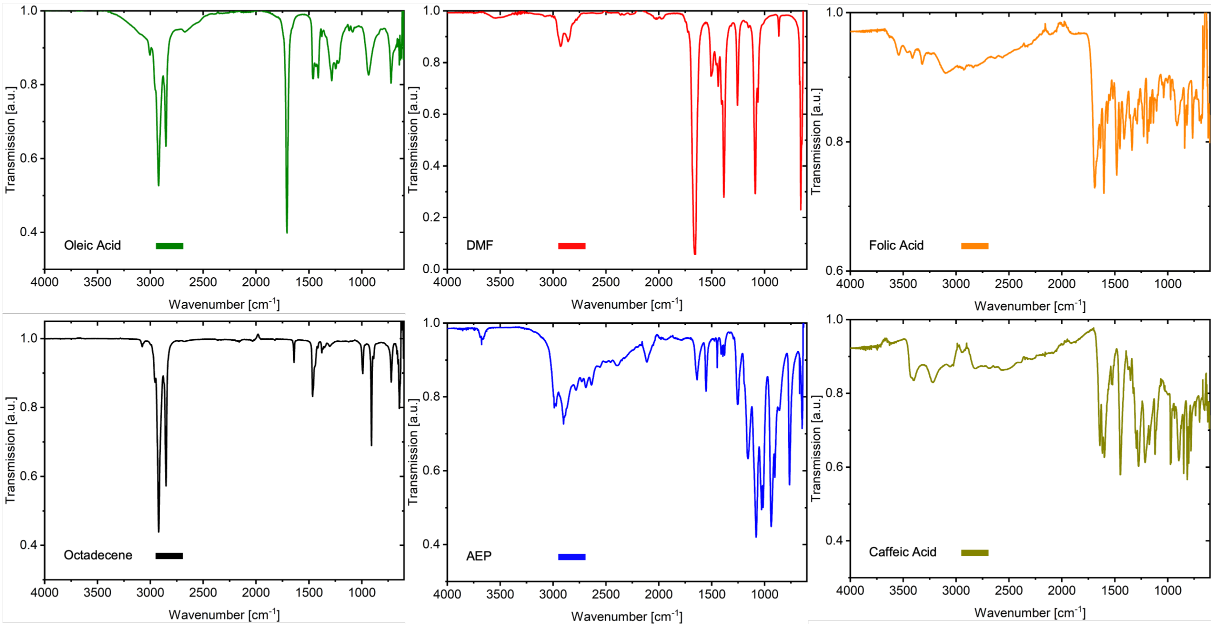

Suppl. Figure S3: FTIR analysis of solvents and pure reagents involved during the surface functionalization. a.u.: arbitrary units.

FTIR

The FTIR spectrum of the as-prepared core/shell nanoparticles (Figure 3B) showed distinct signals located at 2926 cm^{-1} and 2852 cm^{-1}, which indicated an alkyl component with its ν_{as}(-CH_2-) and ν_s(-CH_2-) vibrations modes. [1] Further, distinct signals at 1541 cm^{-1} and 1460 cm^{-1} were assigned to the $\nu_{as}(COO^-)$ and $\nu_s(COO^-)$ vibration modes of carboxylate groups, indicating a deprotonated terminal carboxylic acid and by this confirming the presence of oleic acid as initial surface capping. [1,2]

Subsequently, nitrosyl tetrafluoroborate ($NOBF_4$) was used to substitute oleic acid and provide an easily exchangeable precursor for future modifications in polar media. After the reaction, the oleic acid-attributed vibrations located at 2800 cm^{-1} to 3000 cm^{-1}(-CH_2-) and 1400 cm^{-1} to 1600 cm^{-1}(COO^-) vanished, confirming the removal of oleic acid from the particle's surface. Novel vibrations emerged at 1666 cm^{-1} and 1390 cm^{-1}, which were found to originate from the ν(C=O) and $\delta_s(CH_3)$N of the dimethylformamide's (DMF) amide (see Suppl. Figure S3). [3,4] This coordination of solvent molecules on the particle surface was in agreement with literature reports for this procedure. [4] Vibrations of the tetrafluoroborate anion (BF_4^-) could not be unambiguously assigned due to the interfering signals of DMF around ≈1000 cm^{-1} to 1100 cm^{-1}.

After transfer into a polar medium, 2-aminoethyl dihydrogen phosphate (AEP) as terminal amine-bearing linker

was introduced. The FTIR spectrum revealed the loss of previously dominant DMF vibrations and showed new vibrations located around 1300-900 cm^{-1}. The complex signal was matched to ν(P—O) vibrations of the AEP's phosphate group (PO_4^{3-}) and was in accordance with literature reports.[5,6] Apart from the particle-coordinating phosphate, and the small alkyl constituent indicated by the vibrations between 3000 - 2800 cm^{-1} and 1456 cm^{-1}, the vibration at 1554 cm^{-1} was assigned to a terminal amine.[7–9]

Enabled by the terminal amine, the particles that were functionalized using FA that revealed a characteristic amide bond peak at 1649 cm^{-1}.[10,11] Additionally, the signals located at 1606 cm^{-1} and 1510 cm^{-1} were assignable to vibrations related to the phenyl and pterin rings of FA.[12] Moreover, the bending vibration of the terminal amine, located at 1554 cm^{-1}, disappeared after FA functionalization. In comparison to this, the CA functionalization showed a more prominent vibration at 1649 cm^{-1} and a weaker one at 1537 cm^{-1}. These were assigned to the amide I and II bands, respectively.[11,13] In addition, two shoulders were detected in the broad signal between 3500 - 2800 cm^{-1}, which were characteristic for O—H stretching vibrations of the catechol moiety of caffeic acid.[14]

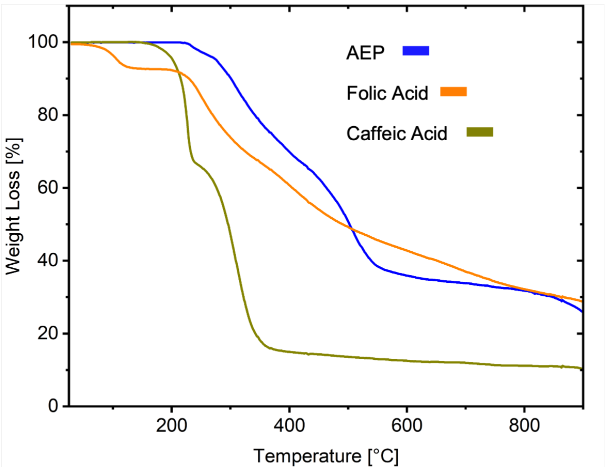

Suppl. Figure S4: TGA analysis of the pure surface modifications, used for surface functionalization.

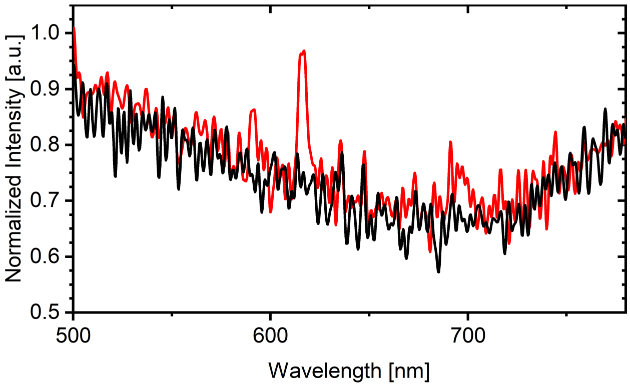

Suppl. Figure S5: CL spectrum of europium-doped nanoparticles after simple data processing; 0.366157 Hz Low Pass filter applied. a.u.: arbitrary units.

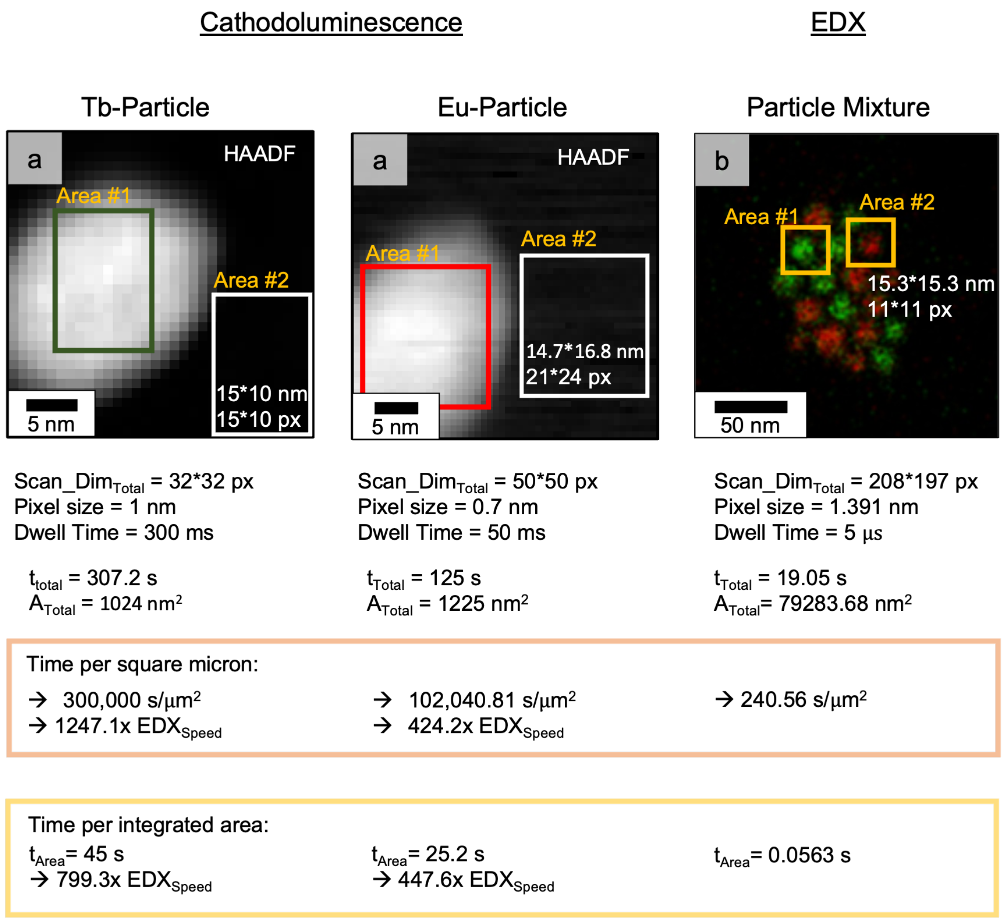

Suppl. Figure S6: Comparison of acquisition times used during single-particle analysis. Particularly emphasised are the t/μ^2 (orange) and the time per target (yellow), where the relationships between the experiments are shown in the tables.

The comparison of CL and EDX-SM in Suppl. Figure S6 shows the striking differences in acquisition speed. The CL images were acquired using a higher resolution with a smaller pixel size, however, EDX-SM also offered a resolution suitable for ultrastructural assessment of biological samples. Given a significantly larger area mapped, while requiring a fraction of the time needed for the CL analysis, the potential of EDX-SM is clearly demonstrated. Even if CL is given the benefit of the doubt since the acquisition parameters are not completely consistent, the times per area are of such different magnitudes that EDX-SM showed its advantages, especially when taking into account that within its acquisition time, EDX-SM offered superior data quality. It was therefore assumed that EDX could be assigned a superiority unattainable for CL within this STEM set-up.

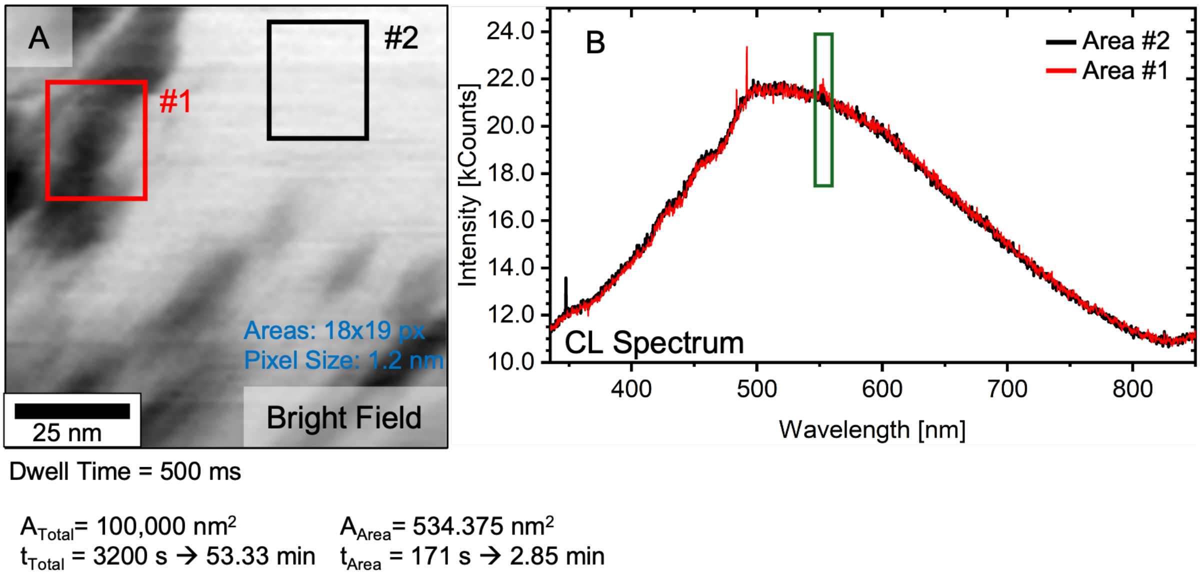

Suppl. Figure S7: (**A**) TEM bright field image of epoxy-embedded terbium-doped nanoparticles. CL spectrum, showing the particle's CL response integrated across area #1 and #2, location shown in (**B**). Expected electronic transition indicated using area; Tb3+-doped (green).

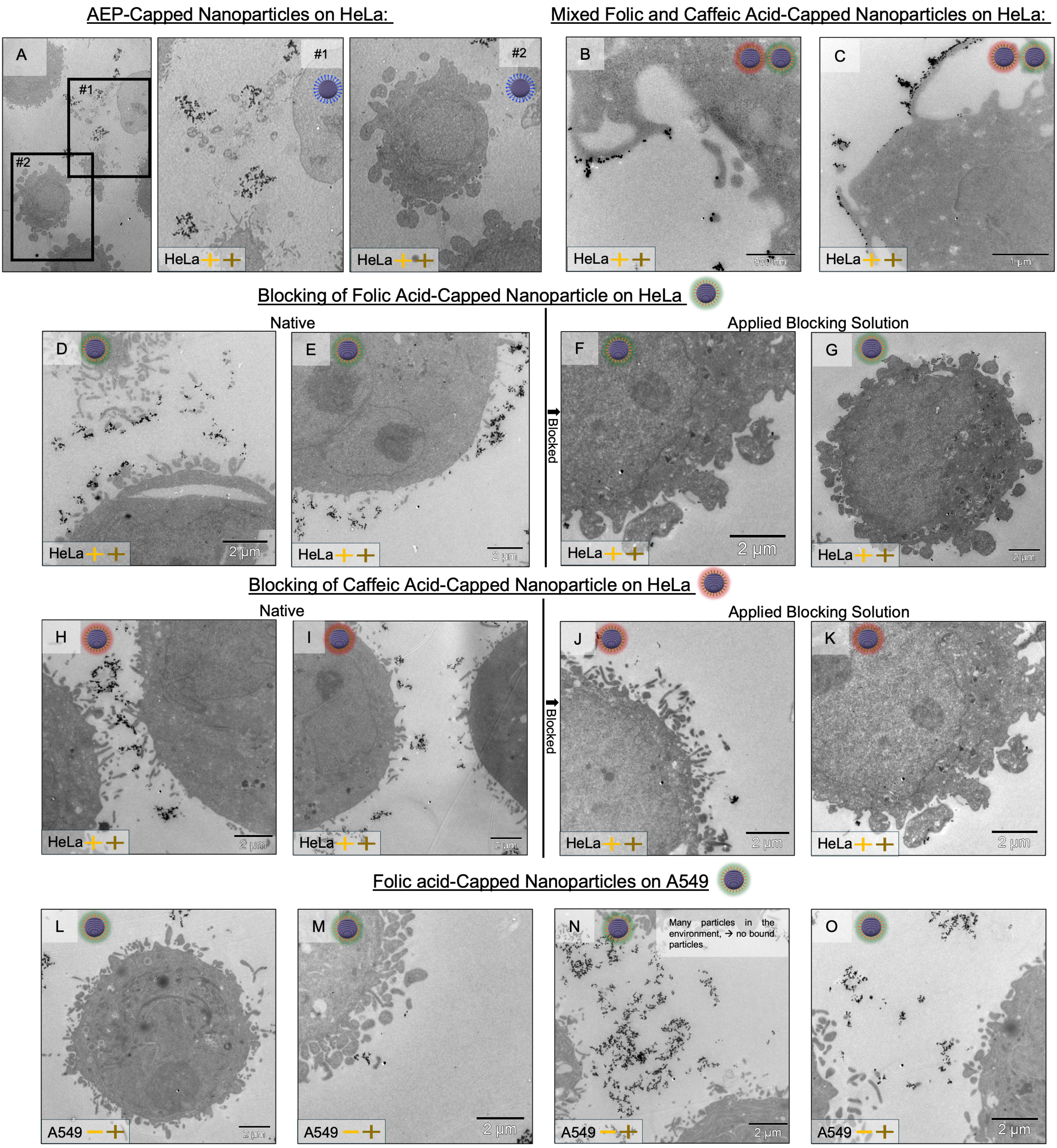

Suppl. Figure S8: Cell Labelling and Blocking Experiments. Representative TEM bright-field images of 80-100 nm-sections deposited on formvar grids showing binding behaviour of nanoparticles under different labelling conditions: (**A**) Non-ligand functionalized, AEP-capped nanoparticles on HeLa cells; (**B+C**) Mixture of folic acid and caffeic acid-capped nanoparticles on HeLa cells; (**D-G**) Folic acid-capped nanoparticles on HeLa cells without blocking solution (**D+E**) and with exposure to a folic acid-containing blocking solution (**F+G**); (**H-K**) Caffeic acid-capped nanoparticles on HeLa cells without blocking solution (**H+I**) and with exposure to a caffeic acid-containing blocking solution (**J+K**); (**L-O**) Folic acid-capped nanoparticles on A549 cells (folate receptor-negative cell line. Detailed description of the blocking experiments can be found in section 9.3.5. Receptor expression indicated using +/-; yellow: folate receptor, brown: β-adrenergic receptor.

References

[1] T. Cao, T. Yang, Y. Gao, Y. Yang, H. Hu, F. Li, *Inorganic Chemistry Communications* **2010**, *13*, 392–394.

[2] K. Yang, H. Peng, Y. Wen, N. Li, *Applied Surface Science* **2010**, *256*, 3093–3097.

[3] V. Venkata Chalapathi, K. Venkata Ramiah, *Proc. Indian Acad. Sci.* **1968**, *68*, 109–122.

[4] A. Dong, X. Ye, J. Chen, Y. Kang, T. Gordon, J. M. Kikkawa, C. B. Murray, *J. Am. Chem. Soc.* **2011**, *133*, 998–1006.

[5] A. Nsubuga, M. Sgarzi, K. Zarschler, M. Kubeil, R. Hübner, R. Steudtner, B. Graham, T. Joshi, H. Stephan, *Dalton Trans.* **2018**, *47*, 8595–8604.

[6] J. Y. Chane-Ching, A. Lebugle, I. Rousselot, A. Pourpoint, F. Pellé, *J. Mater. Chem.* **2007**, *17*, 2904–2913.

[7] K. Liu, X. Liu, Q. Zeng, Y. Zhang, L. Tu, T. Liu, X. Kong, Y. Wang, F. Cao, S. A. G. Lambrechts, M. C. G. Aalders, H. Zhang, *ACS Nano* **2012**, *6*, 4054–4062.

[8] F. Ai, Q. Ju, X. Zhang, X. Chen, F. Wang, G. Zhu, *Sci Rep* **2015**, *5*, 10785.

[9] G. Velazquez, A. Herrera-Gómez, M. O. Martın-Polo, *Journal of Food Engineering* **2003**, *59*, 79–84.

[10] X. Zhao, J. Zhang, L. Shi, M. Xian, C. Dong, S. Shuang, *RSC Adv.* **2017**, *7*, 42159–42167.

[11] C. Berthomieu, R. Hienerwadel, *Photosynth Res* **2009**, *101*, 157–170.

[12] Y. Hu, B. Wu, Q. Jin, X. Wang, Y. Li, Y. Sun, J. Huo, X. Zhao, *Talanta* **2016**, *152*, 504–512.

[13] J. Tošović, *Kragujevac Journal of Science* **2017**, 99–108.

[14] Y. Wang, A. Pitto-Barry, A. Habtemariam, I. Romero-Canelon, P. J. Sadler, N. P. E. Barry, *Inorganic Chemistry Frontiers* **2016**, *3*, 1058–1064.
